# Evaluating Feasibility and Durability of the Aero Prosthetic Liner in Transtibial Prosthetic Users

**DOI:** 10.33137/cpoj.v6i1.41865

**Published:** 2023-12-26

**Authors:** Y Miyata, K Sasaki, G Guerra, W Dacharux, P Chaiwan

**Affiliations:** 1 Sirindhorn School of Prosthetics and Orthotics, Faculty of Medicine Siriraj Hospital, Mahidol University, Bangkok, Thailand.; 2 Department of Exercise and Sport Science, St. Mary's University, San Antonio, TX, USA.; 3 Department of Anatomy, Faculty of Medicine Siriraj Hospital, Mahidol University, Bangkok, Thailand.

**Keywords:** Limb Loss, Amputation, Prosthesis, Rehabilitation, Prosthetic, Prosthetic liner, Satisfaction, Comfort, Quality of Life, Questionnaire, Socket Comfort

## Abstract

**BACKGROUND:**

The choice of prosthetic socket interface material significantly affects user comfort and satisfaction. The Affordable Ethylene-Vinyl Acetate Roll-On (AERO) liner was created with the aim of improving functionality and streamlining the wearing process for users.

**OBJECTIVE:**

The purpose of this study was to comprehensively assess user satisfaction, comfort, and durability of the AERO liner and compare it with the common soft Pe-Lite liner.

**METHODOLOGY:**

Fourteen individuals with transtibial amputation participated in this three-month randomized crossover trial study. The Prosthesis Evaluation Questionnaire (PEQ), Expanded Socket Comfort Score (ESCS), and liner thickness measurements were used to comprehensively compare the AERO and Pe-Lite liner.

**FINDINGS:**

The AERO liner demonstrated notable improvements in prosthetic comfort and functionality over Pe-Lite liner. After three months use, there was a significant reduction in reported frustration with the AERO liner (p=0.023, r=0.604) in the PEQ subscale. Specific aspects, such as walking with the prosthesis (p=0.030, r=0.601) and odor perception (p=0.024, d=0.579), favored the use of the AERO liner. The expanded socket comfort score (ESCS) revealed significant superiority for the AERO liner “at best” (p=0.04) and “on average” (p=0.02) after one and three months, respectively. Liner thickness analysis showed significant reductions at the mid-patellar tendon location for the AERO liner at one (0.57±0.48) and three months (0.90±0.69, p=0.01) and in the posterior region after three months (0.63±0.64, p=0.05).

**CONCLUSION:**

Our study highlights the potential advantages of the AERO liner in enhancing comfort and satisfaction. Yet, durability and thinning of the liner when compared to Pe-Lite may be a concern which may eventually affect socket fit. These findings contribute to ongoing efforts to optimize prosthetic interventions and improve the quality of life of individuals with lower limb prosthesis in resource-limited environments.

## INTRODUCTION

Transtibial amputations are prevalent worldwide, particularly in low and middle-income countries, due to various factors such as diabetes, trauma, and congenital conditions. Prosthetic treatment is vital for improving the satisfaction of people with amputation, and enhancing their quality of life (QoL).^1,2^ However, people with amputation in resource limited environments (RLE) face a range of challenges, encompassing issues of accessibility, constraints related to available resources, and the quality of prosthetic devices.^3^ Rehabilitation success in people with lower limb amputation depends on factors like pre-amputation mobility, time between amputation and prosthetic fitting, and material selection.^4^ The elements taken into consideration for prosthetic intervention include the affordability, fabrication time, safety, and durability of the material. Thus, the selection of appropriate materials for the development of prosthetic devices plays a crucial role in enhancing the QoL for people with amputation.^5^

The effectiveness of interface materials, particularly their impact on the residual limb, is an intriguing area of study. Roll-on gel liners, for instance, have shown higher satisfaction levels compared to polyethylene foam (Pe-Lite).^6^ The ethylene-vinyl acetate (EVA) material exhibits similarities to Pe-Lite because both are closed-cell polymers. EVA is widely used in a multitude of product applications and is acknowledged for its cost-effectiveness.

A cost-effective alternative, referred to as the Affordable Ethylene-Vinyl Acetate Roll-On (AERO) liner, has been created with the aim of improving functionality and streamlining the process of wearing for users.^7^ This prosthetic liner makes use of materials obtained from local sources and features a production method that is simple and easy to follow. Consequently, it provides an economically viable and environmentally friendly alternative for prosthetic liners in locations with limited resources. Preliminary pilot data suggested possible improvements in prosthesis comfort, stability, and pressure distribution in individuals with transtibial amputations. Nevertheless, it is imperative to recognize the constraints arising from a limited sample size and the selection of participants with optimal residual limb configurations.^8^ Therefore, assessing the liner's long-term impact and considering the user experience are crucial factors in revealing the strengths and weaknesses of the AERO liner.^9^ It was hypothesized that for individuals using the AERO liner prosthesis, comfort across a three-month use period would be maintained. Thus, our study purpose was to explore utility of the AERO liner in prosthesis users during a three-month period.

## METHODOLOGY

### Participants

This study was approved by the Siriraj Faculty of Medicine Institutional Review Board (Si 419/2022). This study involved the participation of thirteen people with unilateral transtibial amputation and one with bilateral transtibial amputations. Prior to their involvement, all participants provided informed consent.

### Experimental protocol

**Figure 1:**
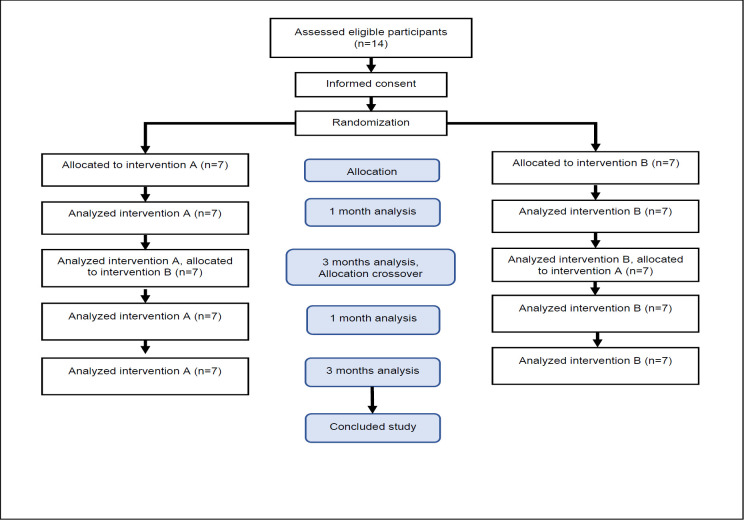
Flowchart of participant allocation during the study, n= number of participants. Note: The number of participants: thirteen with unilateral transtibial amputations and one with bilateral transtibial amputation.

This study utilized a crossover design in which participants were randomly assigned to Pe-Lite or AERO liner groups (**Figure 1**). A certified prosthetist created and fit all the prostheses, which were patella tendon bearing (PTB) socket with Pe-Lite liner, in our clinic. And AERO liners were fit with either a prefabricated or custom-made liner. In total there were fourteen participants. Liners were either prefabricated in small, medium, and large sizes. Or custom made for a unique limb shape. Ten unilateral participants used prefabricated liners, three unilateral participants used custom liners, and finally one bilateral participant used a prefabricated liner on one limb and custom liner on the other limb. Pe-Lite liners were also fabricated specifically for all participants. Both liners were fabricated from a 5 mm thick material. No adjustments were necessary for user of PTB sockets with cuff strap. However, minor adjustments, such as the addition of a pad were made for PTB sockets with supracondylar suspension designs to ensure suspension. The prosthetist also instructed the participants on how to perform the roll-on donning method to ensure consistent and unbiased donning. The AERO liner was provided with two liners per each participant, one designated for primary use and the other as a spare liner. Participants were instructed to change to using the spare liner only if necessary for limb comfort and function. This approach ensured that participants had access to a backup liner in case of wear and tear or other unforeseen circumstances during the study.

The Thai PEQ^10^ was used to evaluate patient satisfaction across different domains. The PEQ, originally developed in English, is composed of 82 questions, which are further divided into 9 subscales. These subscales are Ambulation (AM), Appearance (AP), Frustration (FR), Perceived Response (PR), Residual Limb Health (RL), Social Burden (SB), Sounds (SO), Utility (UT), and Well Being (WB). In this study, we used four subscales: AM, FR, RL, and UT, which are related to interface material evaluation. Additionally, we assessed nine individual items to gain insight into the effect of the liner on the patients. Participants completed the PEQ after one month and three months of using their prosthetic device.

The participant's comfort was assessed using the expanded socket comfort score (ESCS). Traditionally employed straightforward questionnaires may assess prosthetic comfort, however, obtaining feedback from users regarding their experiences with best, worst, and average comfort levels is one approach. This approach offers a more comprehensive evaluation of socket comfort than solely considering current comfort ratings.^11^

**Figure 2:**
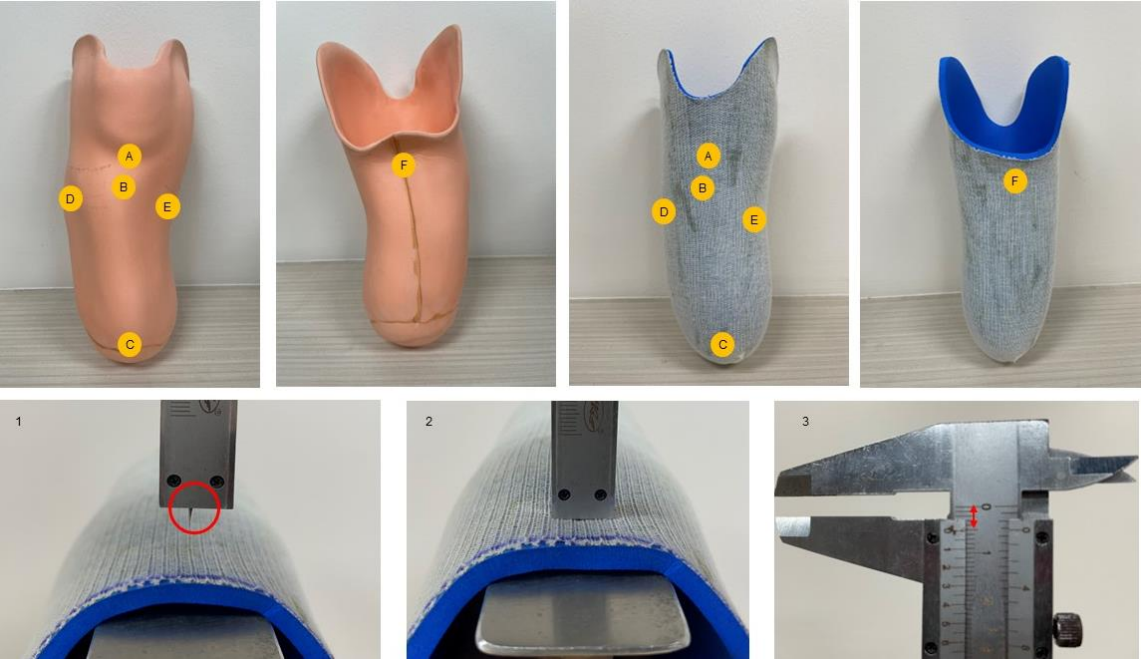
The six areas used for evaluating liner thickness: (A) mid patellar tendon, (B) tibial tubercle, (C) distal end of tibia, (D) head of fibula, (E) medial flare, and (F) posterior. Liner thickness was check using the following procedure; (1) prepare caliper depth gauge and metal bar to sandwich the liner, (2) penetrate liner and check for contact with metal bar, (3) check the thickness of the caliper gauge.

We assessed pressure-sensitive and pressure-tolerant areas, including the mid-patellar tendon (MPT), tibial tubercle, distal end of the tibia, head of the fibula, medial flare, and posterior region. The liner thickness was measured using a depth gauge caliper, which measured the liner material, as shown in **Figure 2**. A caliper depth bar precision of 0.05 mm was utilized to evaluate liner thickness at intervals of 1 month and 3 months for each liner. These three outcome measures, PEQ, ESCS, and liner thickness evaluation, were administered for Pe-Lite and AERO liner prostheses.

### Data analysis

Statistical analysis was conducted using R statistical software version 4.2.0 (R Project for Statistical Computing). To analyze the performance on the PEQ and ESCS between Pe-Lite and AERO liner at one month and three months, a Wilcoxon signed Rank Test (p < 0.05) was used. The designation “NR” for non-responses, which signified the absence of pain associated with the device, was evaluated using the Mann-Whitney U Test. The PEQ effect size was calculated using r, with interpretation based on established criteria: d > 0.10 denoting a small effect, d > 0.30 indicating a medium effect, and d > 0.50 signifying a large effect, depending on the statistical significance of the findings. The liner thickness for both Pe-Lite and AERO liner was analyzed using a Student's t-test for one and three-month.

## RESULTS

**Figure 3:**
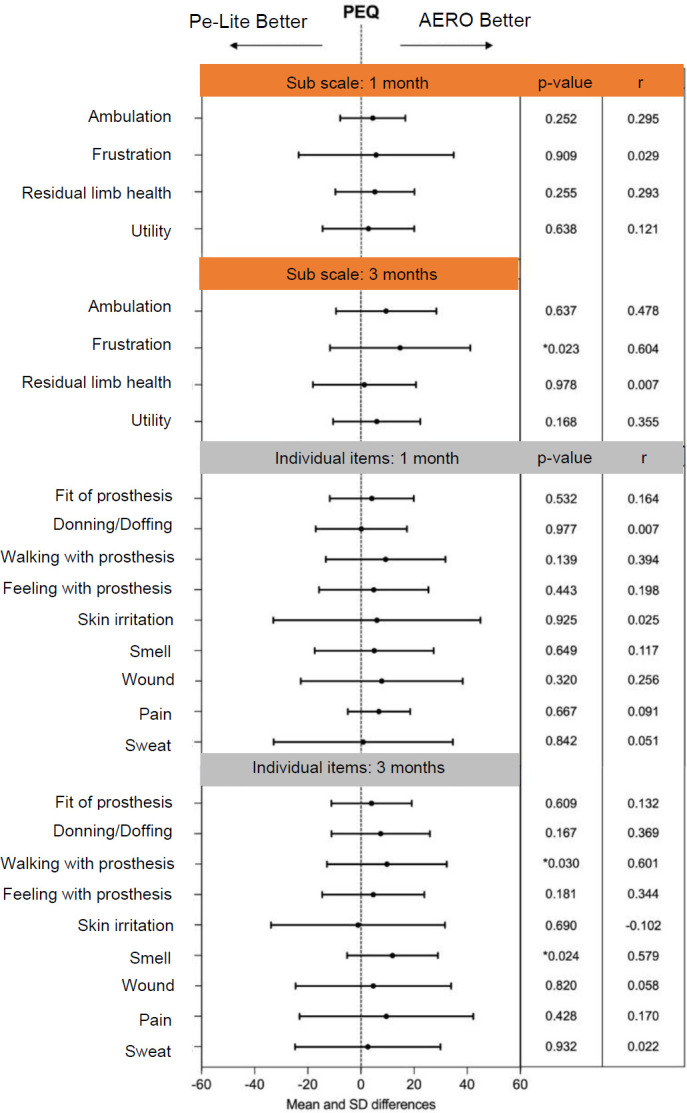
Prosthesis evaluation questionnaire per subscale and individual items at one and three months for Pe-Lite and AERO liner. * Indicates significant differences (p<0.05).

**Figure 4:**
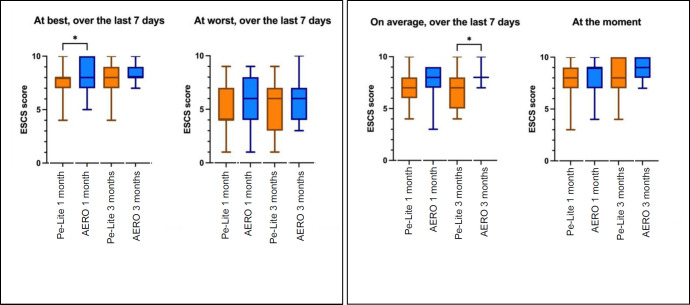
Expanded socket comfort score results at one and three months for PE-lite and AERO liner. * Indicates significant differences (p<0.05).

**Figure 5:**
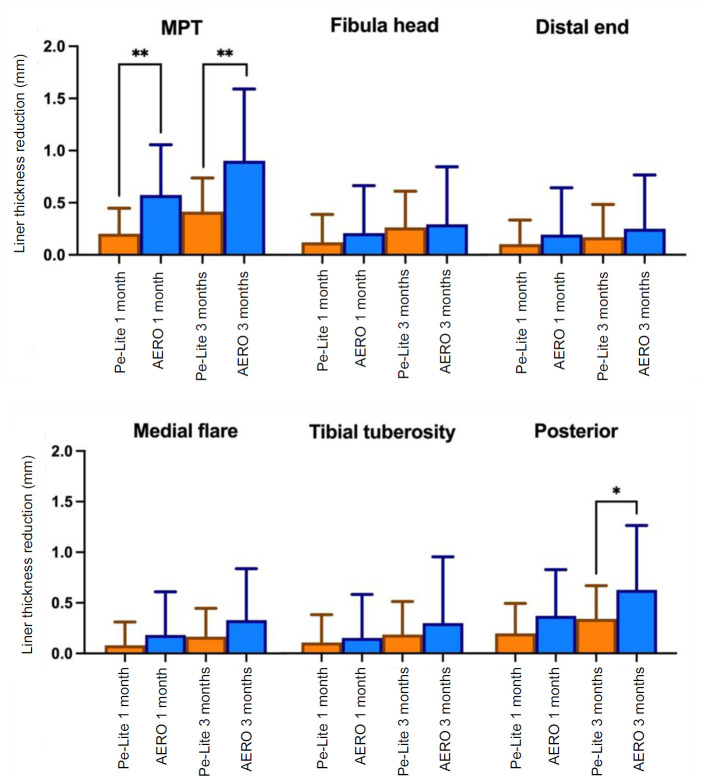
Liner thickness differences over time between the one month and three-month intervals. These changes are analyzed across six regions: MPT; mid patellar tendon, fibula head, distal end of tibia, medial flare, tibial tuberosity and posterior. * Indicates significant differences (p<0.05), and **(p<0.01).

This study involved fourteen participants, thirteen with unilateral transtibial amputations and one with bilateral transtibial amputation. The participants included twelve males with an average age of 52.1±10.4 years, and two females with an average age of 59.5±0.7 years. All participants used PTB prosthetic sockets, with eight using a cuff strap suspension system, including one person with bilateral transtibial amputation and six using an anatomical supracondylar suspension. Analysis of the PEQ revealed a significant reduction in reported frustration after three months with the AERO liner (p=0.023, r=0.604). Additionally, specific items related to walking with the prosthesis (p=0.030, r=0.601) and odor perception (p=0.024, d=0.579) indicated better results with the AERO liner compared to Pe-Lite after three months. While participants generally expressed a preference for the AERO liner, most preferences did not reach statistical significance compared to Pe-Lite (**Figure 3**). Three participants in the Pe-Lite group indicated “NR” (no pain at the residual limb) at both one month and three-month assessments. In contrast, within the AERO liner group, four participants reported “NR” after one month, and this number increased to five participants after three months (**Figure 3**). The result of the ESCS for the AERO liner, showed significantly superior results when compared to Pe-Lite “at best, over the last days”, 8.0±1.6 (p=0.04) after one month and “on average, over the last days”, 8.1±0.9 (p=0.02) after three months (**Figure 4**). A significant reduction in liner thickness was observed at the MPT location for the AERO liner compared to Pe-Lite, both after one month 0.57±0.48 and three months 0.90±0.69 follow-up (p=0.01). Additionally, a significant difference was noted in the posterior region after three months 0.63±0.64 (p=0.05) (**Figure 5**).

## DISCUSSION

We investigated the satisfaction, comfort, and changes in liner thickness of transtibial prosthetic users during three-month use of AERO and Pe-Lite liner. The findings of this study shed light on several crucial aspects of transtibial prosthetic use and, in particular, the impact of the AERO liner on user experience. Our results demonstrated a significant reduction in reported frustration after three months of utilizing the AERO liner compared to Pe-Lite liner. Participants expressed a preference for the AERO liner, although this preference did not reach statistical significance when compared with Pe-Lite liner. These findings suggest that the AERO liner offers notable improvement in terms of user experience, particularly with regard to frustration reduction. Frustration reduction can play a crucial role in enhancing overall prosthetic satisfaction, as users are more likely to continue using devices that minimize daily challenges and discomfort.^12,13^ Although the preference for the AERO liner did not reach statistical significance, it is possible that with a larger sample size, this trend might become more pronounced. Therefore, future research with a larger participant pool may provide further insights into the preference of the AERO liner over the Pe-Lite liner.

Pain is a significant concern for individuals using prosthetic devices as it can affect mobility, quality of life, and long-term compliance with prosthetic use.^14-16^ Three participants reported “NR” (no pain in the residual limb) when using Pe-Lite after one and three months. In contrast, in the AERO liner group, four participants reported “NR” after one month, and this number increased to five participants after three months. Roll-on donning of the AERO liner may permit accommodation to the residual limb surface, and liner softness may reduce pain. Although differences were minimal, these findings suggest that the AERO liner might have contributed to a slightly reduced pain perception in users.

Prosthetists often express concern about liner durability when prescribing devices.^5^ Liner thickness is a critical factor for prosthetic comfort and fit. In our analysis, we observed significant differences in the liner thickness changes over time, particularly at the MPT location. The AERO liner demonstrated a greater reduction in liner thickness at both one month and three months compared to the Pe-Lite liner. Additionally, a significant reduction in liner thickness was noted in the proximal posterior region after three months. The softer AERO liner, which has more flexible material properties, may contribute to the observed reduction in liner thickness. Also, the observed outcome is attributed to the proximal pressure exerted on the AERO liner. Prolonged exposure to such pressures could potentially compromise the liner's durability, leading to discomfort within the prosthesis. Therefore, it is essential to consider the shape of the prosthetic socket to avoid localized pressure.

One approach to minimize the impact of these symptoms is socket design. In this study, all participants used the PTB socket, which is a common choice for RLE. The PTB socket was designed to provide proximal compression within the socket for weight bearing. However, concerns have been raised in some studies about the potential impact of this socket design on residual limb.^17,18^ Given the roll-on application method of the AERO liner, it may be worth exploring the suitability of total surface bearing socket (TSB) to enhanced comfort and load distribution.^19-21^ Evenly distributing pressure across the liner is believed to reduce localized pressure points and maintain consistent liner thickness.^22,23^

### Limitations

The limitations of this study must be acknowledged. The relatively small sample size may have influenced the statistical significance of certain findings such as user preferences. Since participants had the option to change to their AERO spare liner if necessary, this may have had an effect on satisfaction and odor. Future research, with larger and more diverse participant pools, may provide additional insights. Outcomes of interest in this study was comfort and satisfaction, which are pivotal factors that influence the acceptance and long-term use of prosthetic devices. Previous studies have recognized the effectiveness of gel liners but have also highlighted concerns related to discomfort resulting from sweating.^6^ While the AERO liner facilitates roll-on donning for a better fit with the residual limb, our investigation did not reveal any significant evidence supporting the notion that AERO liner usage is linked to increased perspiration.

## CONCLUSION

In conclusion, this study highlights a potential comfort and satisfaction benefit of using the AERO liner. However, proximal liner thickness may reduce over time which might impact socket fit and comfort. While our results indicate promising trends, further research is needed to confirm these findings and explore the interplay between liner types, socket shapes, and user experiences comprehensively. These findings contribute to ongoing efforts to enhance the quality of life of individuals with lower limb amputations who reside in RLE by optimizing prosthetic interventions.

## DECLARATION OF CONFLICTING INTERESTS

The authors declare no conflicts of commercial or financial interest in this research.

## AUTHORS CONTRIBUTION

- **Yusuke Miyata:** original drafting, writing, data collection, statistical analysis.
- **Kazuhiko Sasaki:** conceptualization, writing, data collection, statistical analysis.
- **Gary Guerra:** writing, statistical analysis, proofing.
- **Woratee Dacharux:** writing, data collection, proofing.
- **Pilipda Chaiwan:** writing, data collection, proofing.

## SOURCES OF SUPPORT

This research received funding from the Faculty of Medicine, Siriraj Hospital, Mahidol University.

## ETHICAL APPROVAL

This study was approved by the Siriraj Faculty of Medicine Institutional Review Board (Si 419/2022).
